# Trends in Emergency Department Visits and Surgeries due to Traumatic Brain Injury During the COVID-19 Pandemic in Finland

**DOI:** 10.1007/s42399-023-01437-9

**Published:** 2023-03-14

**Authors:** Saara Jäntti, Ville Ponkilainen, Ilari Kuitunen, Mikko Uimonen, Teemu Luoto, Ville M. Mattila

**Affiliations:** 1 Faculty of Medicine and Health Technology, Tampere University, Kauppi Campus, Arvo Ylpön Katu 34, 33520 Tampere, Finland; 2 Department of Surgery, Central Finland Hospital Nova, Hoitajantie 3, 40620 Jyväskylä, Finland; 3 Institute of Clinical Medicine, University of Eastern Finland, Yliopistonranta 1, 70211 Kuopio, Finland; 4 Department of Pediatrics, Mikkeli Central Hospital, Porrassalmenkatu 35-37, 50100 Mikkeli, Finland; 5 Department of Neurosurgery, Tampere University Hospital, PL 2000, 33521 Tampere, Finland; 6 Department of Orthopaedics and Traumatology, Tampere University Hospital, Teiskontie 35, PL 2000, 33521 Tampere, Finland; 7 Coxa Joint Replacement Hospital, Niveltie 4, 33520 Tampere, Finland

**Keywords:** Traumatic brain injury, COVID-19, Neurosurgery, Craniotomy

## Abstract

We aim to evaluate the changes in the incidence of TBI, trauma craniotomies, and craniectomies during the COVID-19 pandemic in Finland. This retrospective register study was conducted at three Finnish hospitals. We retrieved the numbers of emergency department (ED) visits, inpatient admissions, and trauma craniotomies and craniectomies due to TBI in the adult population from 2017 to 2020.We calculated the incidences per 100 000 inhabitants and compared the year 2020 to the reference years (2017–2019) by incidence rate ratios (IRR) with 95% confidence intervals. The incidence of TBI-related ED visits during the study period compared to the reference years started to decrease in March 2020 (IRR 0.86, CI: 0.73–1.02), and the lowest incidence was seen in April 2020 (IRR 0.83, CI: 0.68–1.01). The incidence of ED visits showed a second decrease in December (IRR 0.80, CI: 0.67–0.96). The incidence of concussion decreased during the national lockdown in March (IRR 0.80, CI 0.66–0.97). The incidence of ED visits due to TBI decreased after the declaration of national lockdown in spring 2020 and showed a second decrease during regional restrictions in December. In addition, the incidence of neurosurgically treated TBI decreased during restaurant restrictions in the spring.

## Introduction

Since the first outbreak of COVID-19 in 2020, elective operations have been regularly postponed and healthcare staff relocated to emergency units in preparation for a possible surge in COVID-19 cases. Following a rapid decrease in the number of COVID-19 cases in May 2020, lockdown restrictions in Finland were lifted at the beginning of June.

Subsequently, a second wave of COVID-19 infections began in the fall of 2020. Although the weekly number of COVID-19 cases during the second wave was higher than in the first, a national lockdown was not initiated, and targeted regional restrictions were implemented instead. The epidemiologic situation was divided into three levels: base level, accelerating level, and spreading level. During the first wave of the COVID-19 pandemic in Finland, restaurants were ordered to close from April to the end of the May 2020. However, restrictions on opening hours and the number of customers allowed in restaurants were still in force during the second wave of the pandemic [1]. During the restrictions, the consumption of alcohol shifted from bars and restaurants to the home, and the volumes of alcohol sold in bars and restaurants decreased. Thus, when compared to alcohol consumption in 2019, a decrease in total alcohol consumption was observed in 2020 [2].

Traumatic brain injury (TBI) is one of the most common causes for emergency department (ED) admissions [3]. Because an individual is more likely to suffer a head trauma during acute alcohol intoxication, alcohol misuse is a major risk factor for TBI [4]. During the first wave of the COVID-19 pandemic, a decrease in the number of emergency referrals due to TBI was reported by several studies. Travel restrictions, social distancing, cancellation of sport activities, and recommendations to work from home caused a decrease in the rate of accidents since people spent more time at home and, for example, traffic road accidents decreased. These types of accidents are usually at high energy and likely to cause head trauma. Citizens may have also avoided unnecessary ED visits, and patients with mild TBI or concussion may have stayed at home instead of seeking medical care [5–9]. However, some studies also reported that the number of TBI patients admitted to ED units remained the same during the pandemic [10]. Within neurosurgery, however, operating volumes decreased worldwide during the first wave of the COVID-19 pandemic [11–13]. A similar trend was also seen in the number of emergency neurosurgeries [14].

Globally, the COVID-19 pandemic, together with the restrictive measures enacted to prevent the spread of the virus, has had an immense impact on the way of life of many people, resulting in numerous changes in social behavior. For example, many people may now be reluctant to seek medical care due to fear of being exposed to the COVID-19 virus. Moreover, these behavioral changes could fundamentally alter the dynamics of emergency care and further increase the overall risk for sustaining injuries such as TBI. In this study, we evaluate the incidences of TBI, trauma craniotomies, and craniectomies during the first and second waves of the COVID-19 pandemic in Finland.

## Material and Methods

This study was conducted at three large Finnish public hospitals. The three hospitals—Tampere University Hospital (tertiary level unit), Mikkeli Central Hospital (secondary level unit with integrated primary care ED), and Central Finland Hospital (secondary level unit with integrated primary care ED)—cover a catchment population of approximately 900,000 inhabitants [15]. The number of ED visits due to TBI in the adult population (18 years or older) was retrieved from the patient information system of the participating hospitals using the International Classification of Diseases 10th Revision (ICD-10) [16] diagnostic codes for TBI: S06.0, S06.1, S06.2, S06.3, S06.4, S06.5, S06.6, S06.7, S06.8, and S06.9. All adult patients who were admitted to the participating hospitals with TBI in 2020, and the years 2017 through 2019 (reference years) were included.

Information on the number of trauma craniotomies and craniectomies (delay of less than 14 days) during 2020, and the reference years were retrospectively retrieved and confirmed from the electronic medical record systems of the participating hospitals using NOMESCO Classification of Surgical Procedures (NCSP) [17] procedure codes (Finnish version). The codes included were AAD00, AAD05, AAD15, and AAK80 combined with the diagnostic code S06*. Only craniotomies and craniectomies due to trauma were included.

Incidences with 95% confidence intervals (CI) were calculated per 100 000 inhabitants by Poisson exact method [18]. The reference population for incidences was calculated using the mean of annual incidence during the years 2017–2019. The crude incidences were compared by incidence rate ratios (IRR) with 95% CI. The statistical analyses were performed using R version 3.6.2 (R Foundation for Statistical Computing, Vienna, Austria).

## Results

**Fig. 1:**
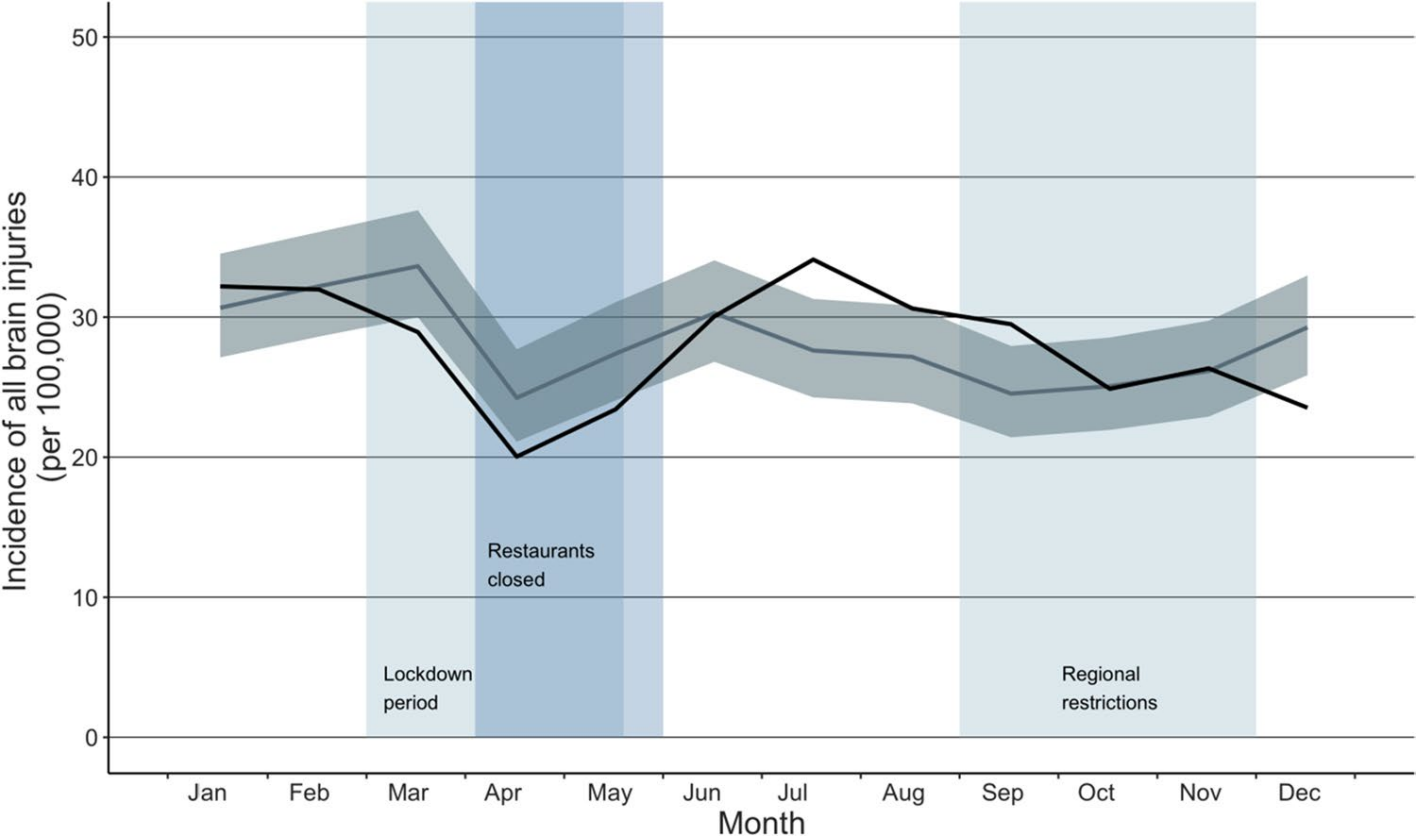
Incidence of all visits due to TBI during the COVID-19 pandemic. The dark line illustrates the incidence during the study period (2020), and the lighter line illustrates the mean of incidences in the reference years (2017–2019) with confidence intervals

The total number of ED visits due to TBI during the years 2017 through 2020 was 11 982, and during the reference years (2017–2019), the mean number of visits due to TBI was 3000. In the participating hospitals, a total of 2981 visits due to TBI occurred during 2020. However, after the declaration of a national lockdown in spring 2020, the incidence of ED visits due to TBI decreased (shown in Fig. 1). The incidence of ED visits during the study period compared to the reference years started to decrease in March 2020 (IRR 0.86, CI: 0.73–1.02), and the lowest incidence was seen in April 2020 (IRR 0.83, CI: 0.68–1.01). Once the lockdown ended in June, however, the incidence of ED visits rebounded to its previous level. A second decrease in the incidence of ED visits occurred in December after targeted regional restrictions were implemented (IRR 0.80, CI: 0.67–0.96). Percentual change between the highest IRR in July (IRR 1.26, CI: 1.05–1.50) and the lowest IRR in December (IRR 0.80, CI: 0.66–0.98) was 36% (shown in Table 1).

**Table 1:** Incidence of ED visits due to TBI during the study period (2020) and reference years (2017–2019)

	Study period (2020)		Reference years (2017–2019)				
Month	*N*	Incidence	*N*	Incidence	IRR	Confidence interval	
Jan	256	28.8	234	26.4	1.09	0.92	1.30
Feb	257	28.9	261	29.5	0.98	0.83	1.17
Mar	236	26.6	275	31.0	0.86	0.72	1.02
Apr	161	18.1	187	21.0	0.86	0.70	1.06
May	184	20.7	215	24.2	0.86	0.70	1.04
Jun	246	27.7	234	26.4	1.05	0.88	1.25
Jul	278	31.3	221	24.9	1.26	1.05	1.50
Aug	248	27.9	213	24.0	1.16	0.97	1.39
Sep	237	26.7	191	21.5	1.24	1.03	1.50
Oct	202	22.7	195	22.0	1.03	0.85	1.26
Nov	213	24.0	209	23.6	1.02	0.84	1.23
Dec	182	20.5	226	25.5	0.80	0.66	0.98

The most common ICD-10 coded reasons for admission to ED units due to TBI were S06.0 (concussion) and S065 (traumatic subdural hemorrhage). During the years 2017 through 2020, the total number of concussions was 9468, and the total number of traumatic subdural hemorrhage was 1713. During the national lockdown, the incidence of concussion, the most common reason for admission to an ED unit due to TBI, decreased, being at the same level as previous years in February (IRR 0.97, CI: 0.81–1.17). Subsequently, the incidence decreased in March (IRR 0.80, CI: 0.66–0.97) and remained lower until May. During the second wave of the pandemic, the incidence of concussion remained at the same level as in the previous years until December (IRR 0.80, CI: 0.65–0.98). Furthermore, the incidence of the other ICD-10 coded reasons for admission to an ED unit did not change notably.

**Fig. 2:**
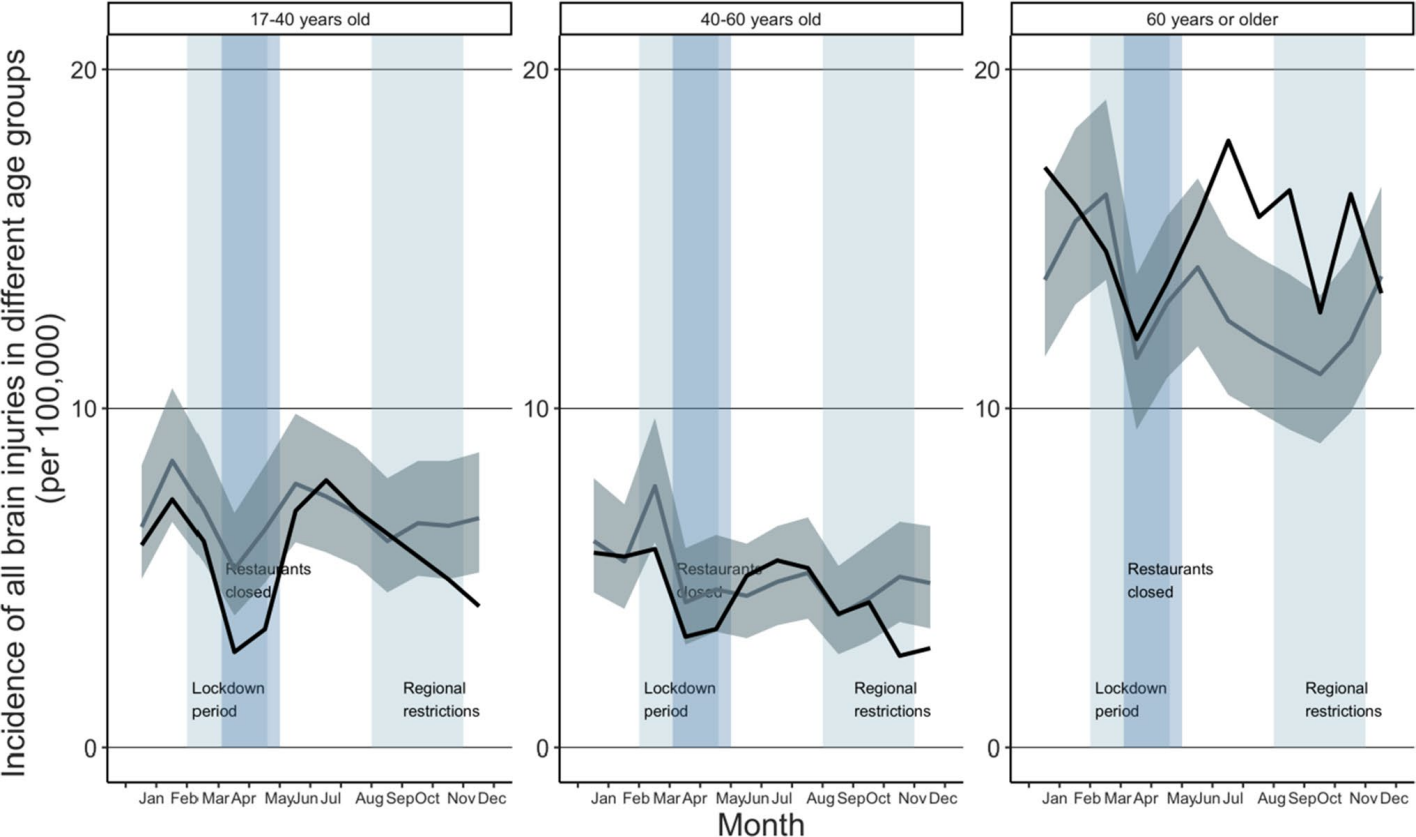
Incidence of ED visits due to TBI in different age groups during the COVID-19 pandemic. The dark line illustrates the incidence during the study period (2020), and the lighter line illustrates the mean of incidences in the reference years (2017–2019) with confidence intervals

**Fig. 3:**
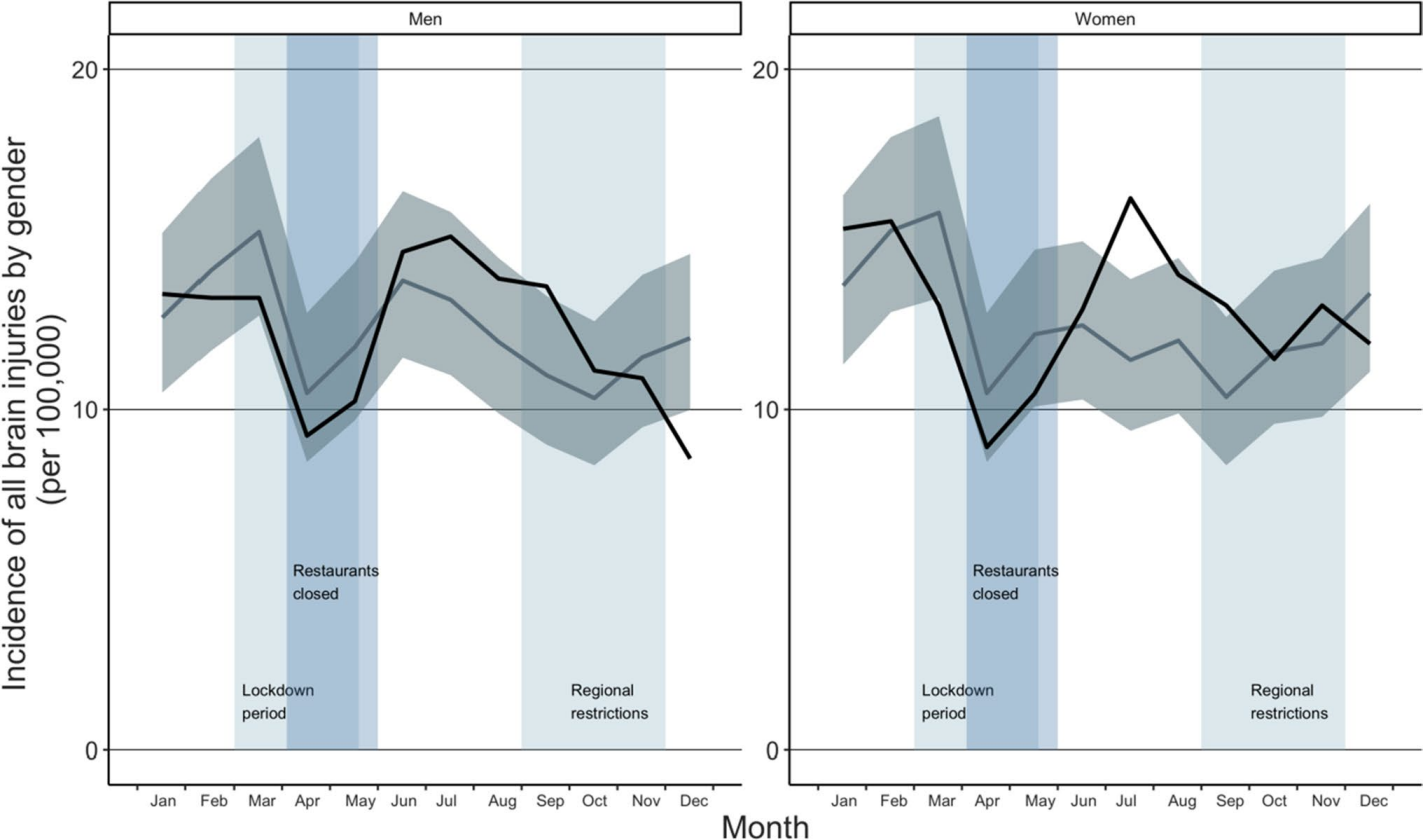
Incidence of ED visits due to TBI by gender during the COVID-19 pandemic. The dark line illustrates the incidence during the study period (2020), and the lighter line illustrates the mean of incidences in the reference years (2017–2019) with confidence intervals

When comparing the incidence of ED visits due to TBI in different age groups, a decrease was seen in age group 17 to 40 years old during the lockdown in April (IRR 0.53, CI: 0.33–0.87) when comparing to previous years (shown in Fig. 2). After the first wave of the pandemic, incidence remained to its previous level being 0.85 (CI: 0.58–1.24) in October. In the age group 40 to 60 years, incidence of ED visits due to TBI remained at the same level as previous years during the year 2020. In elderly (60 years or older), incidence was similar to that in previous years until July when an increase was seen (IRR 1.42, CI: 1.12–1.81). Incidence of ED visits due to TBI remained at higher level than in previous years until December, when it set down to the same level as previous years (IRR 0.96, CI: 0.75–1.24). When comparing ED visits by gender, the incidence did not change notably to that in previous years in men, being 0.87 (CI: 0.65–1.15) in May and 1.08 (CI: 0.81–1.43) in October (shown in Fig. 3). In women, incidence was similar to that in previous years until an increase was seen in July (IRR 1.41, CI: 1.10–1.82). After that, incidence remained the same level than previous years, being 0.98 (CI: 0.75–1.29) in October.

**Fig. 4:**
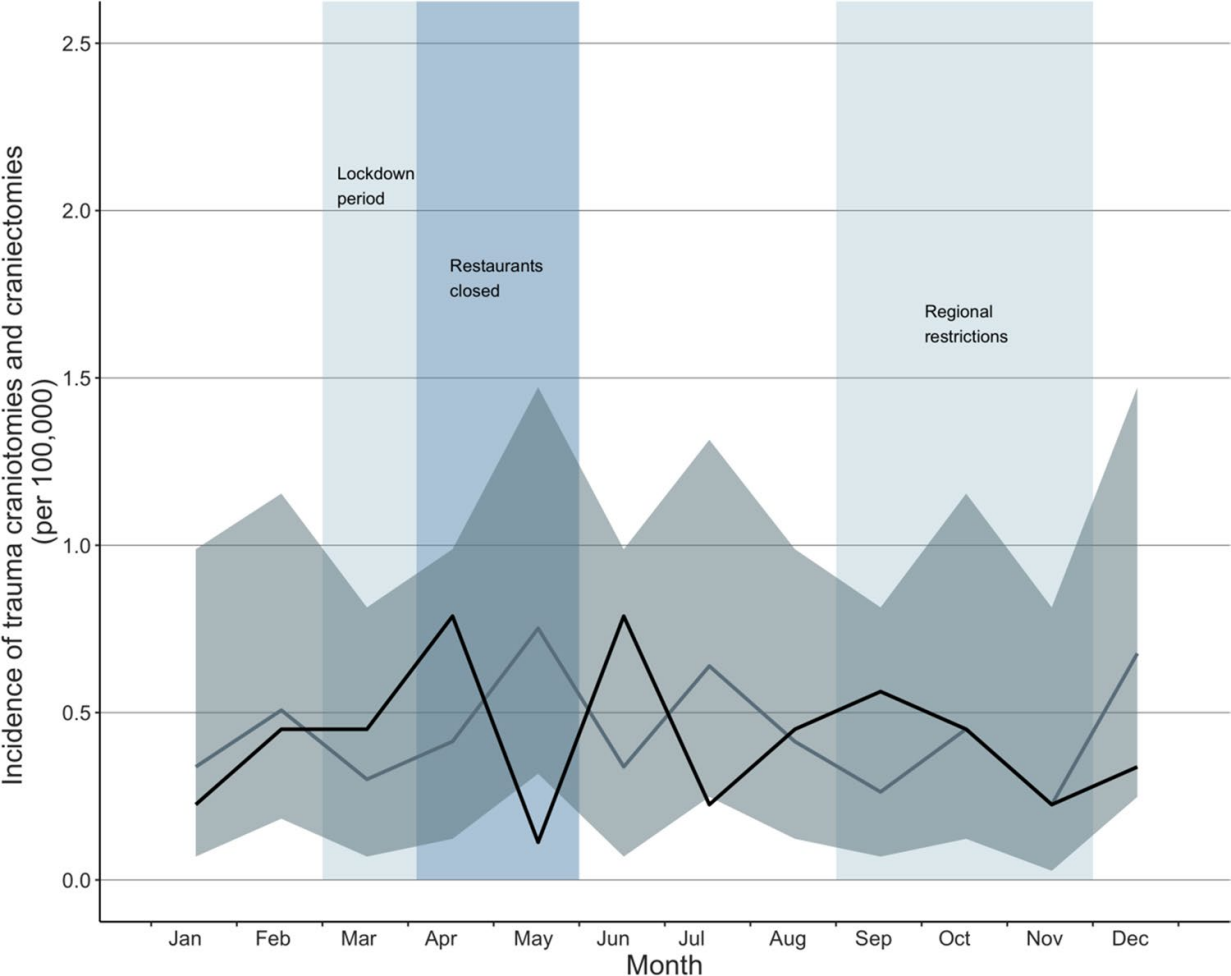
Incidence of trauma craniotomies and craniectomies during the COVID-19 pandemic. The dark line illustrates the incidence during the study period (2020), and the lighter line illustrates the mean of incidences in the reference years (2017–2019) with confidence intervals

The total number of trauma craniotomies and craniectomies during the years 2017 through 2020 was 182. The mean number of operations in the years 2017 through 2019 (reference years) was 46, and the number of operations in 2020 was 45. During the first wave of the COVID-19 pandemic, the incidence of trauma craniotomies and craniectomies was similar to that in previous years until April (IRR 1.90, CI: 0.54–6.75) (shown in Fig. 4). The incidence of trauma craniotomies and craniectomies was lowest in May (IRR 0.15, CI: 0.02–1.23) and rebounded to its previous level in June. During the second wave of the pandemic, the incidence remained at the same level as previous years, being 0.50 (CI 0.12–2.00) in December.

## Discussion

According to the findings of this study, the incidence of ED visits due to TBI decreased after the declaration of a national lockdown in March 2020, with the lowest incidence being observed in April. Thereafter, the incidence rebounded to the same level as in the reference years. This rebound may have been the result of various changes in peoples’ behavior. First, the actual incidence of TBI may have decreased. In addition, most sport and leisure activities were banned during the lockdown, and people were encouraged to work from home. As a result, commuting and traffic volumes decreased, and fewer traffic accidents occurred. Second, citizens were told to avoid unnecessary ED visits, and thus some of the patients with mild TBI/concussion may have avoided seeking medical treatment. These changes may therefore have been some of the main factors behind the changes in incidence rates. Previous studies concerning the first wave of the pandemic have reported similar findings [5–9].

During the second wave of the COVID-19 pandemic in December, the incidence of ED visits due to TBI showed a second decrease. Restaurant and bar restrictions during the national lockdown may have resulted in individuals consuming less alcohol or shifting the place of alcohol consumption from bars to their home. This may, in turn, have led to a decreased incidence of TBIs, as alcohol is a major risk factor for TBI [4].

In the present study, we found that the incidence of trauma craniotomies and craniectomies decreased during the period of restaurant restrictions, being the lowest in May. When the restaurants reopened in June, the incidence of trauma craniotomies and craniectomies rebounded to the same level as in previous years. A decreasing trend in the incidence of trauma craniotomies and craniectomies may be linked to the decrease in the number of TBIs, since fewer TBIs may have led to a reduction in emergency neurosurgery. Indeed, reductions in elective neurosurgery during the COVID-19 pandemic have been reported [11–13, 19], with elective operations canceled or rescheduled to prioritize health care resources and to reduce non-urgent treatment. Similar findings have been reported in emergency neurosurgery [14]. However, according to a previous study from Finland, the nationwide restrictions did not result in a decrease in the number of patients with TBI neurosurgically treated [10].

The strengths of our study include the broad data from three large Finnish hospitals. Furthermore, many previous studies have only evaluated the impact of the first wave of the COVID-19 pandemic. In this study, we were able to collect follow-up data from all patients during the first and second waves of the COVID-19 pandemic and to evaluate the impact of re-opening and restrictions during the second wave. Our current study also has some limitations. Since we aimed to evaluate only trauma patients, we only included specific ICD-10 diagnostic codes. Owing to the retrospective and administrative nature of the present study, we were unable to separately study the different severities of TBI. Moreover, due to the register-based design and uncertainty related to the reliability of ICD-10 and NOMESCO coding, we were only able to evaluate trauma craniotomies and craniectomies, and other neurosurgical traumas were excluded.

In conclusion, the incidence of ED visits due to TBI decreased after the declaration of national lockdown in spring 2020, and a second decrease was observed after the implementation of regional restrictions in December. In addition, the incidence of trauma craniotomies and craniectomies decreased during the restaurant restrictions implemented in the spring. As expected, the COVID-19 pandemic and nationwide restrictions resulted in a decreasing trend in the incidence of ED visits due to TBI and neurosurgically treated TBI.

## Data Availability

Research data are not publicly available due to Finnish research legislation as the Law on the secondary use of routinely collected healthcare data prohibits to share data.
